# Pre-lacteal feeding practice and its associated factors among mothers with children under the age of two years in Dubti town, Afar region, North East Ethiopia: a community based mixed study design

**DOI:** 10.3389/fgwh.2023.1315711

**Published:** 2024-01-09

**Authors:** Temesgen Gebeyehu Wondmeneh

**Affiliations:** Department of Public Health, College of Medical and Health Science, Samara University, Semera, Ethiopia

**Keywords:** pre-lacteal feeding, factors, mothers, children, Ethiopia

## Abstract

**Background:**

Pre-lacteal feeding prevents the early initiation of breastfeeding and exclusive breastfeeding. It is understudied in Afar, a pastoral region in northeast Ethiopia. The study assessed the prevalence of pre-lacteal feeding practice and its associated factors among mothers with children under the age of two years in Dubti, North East Ethiopia.

**Methods:**

A community-based mixed-study design was employed. A systematic sampling technique was used to recruit 370 study participants for the quantitative study, while purposive sampling was used to select 17 study participants for the qualitative study. Logistic regression analysis was used to determine the association between independent variables and dependent variable. The results of logistic regression analysis were presented as an odd ratio with a 95% CI. A *P*-value <0.05 was used as a cutoff point to determine statistical significance. The qualitative data were analyzed using a thematic approach.

**Results:**

Pre-lacteal feeding was practiced by 36% of mothers. Afar ethnicity (AOR = 2.5, 95% CI: 1.2–5.1), an extended family size (≥5) (AOR = 1.73, 95% CI: 1.02–2.9), a birth interval of less than 2 years (AOR = 2.77, 95% CI: 1.59–4.82), the first birth order of an indexed child (AOR = 3.87, 95% CI: 2.14–7.0), male-indexed children (AOR = 2.95, 95% CI: 1.67–5.2), and no antenatal care visits (AOR = 2.67, 95% CI: 1.67), or once or twice antenatal visits were significantly associated with pre-lacteal feeding practice. Breastfeeding counseling (AOR = 0.38, 95% CI: 0.2–0.7) and delivery at a health institution (AOR = 0.3, 95% CI: 0.17–0.55) were protective factors of pre-lacteal feeding practice. The most common pre-lacteal foods were dairy products, water, and certain plant species. Cultural beliefs are the main reason for practicing these pre-lacteal feedings.

**Conclusion:**

A significant number of study participants practiced prelacteal feeding. A public health campaign emphasizing the importance of antenatal care follow-ups should be initiated. Breastfeeding counseling and delivery in a health facility should also be strengthened. Community health education about the disadvantages of pre-lacteal feeding practices should be provided to reduce traditional beliefs.

## Background

Pre-lacteal feeding is defined as the administration of any substance to newborn babies other than breast milk during the first three days after birth (1). Pre-lacteal feeding influences early breastfeeding initiation and exclusive breastfeeding practices (2), which reduces the risk of neonatal mortality (3, 4). Pre-lacteal feeding and prenatal maternal nutrition can have an impact on an infant's growth. Pre-lacteal feeding is associated with diarrhea and lower respiratory tract infections (5). Women play a crucial role in maintaining the health of their families due to their direct contribution to family nutrition (6). Inadequate prenatal maternal nutrition has an impact on the developing fetus and may have a long-term impact on the health and development of the child (7). The World Health Organization (WHO) recommends early and exclusive breastfeeding, which can save over 800,000 lives in children under the age of five years worldwide each year (8). The magnitude of pre-lacteal feeding was 40.1% in Nepal (9) and 56.5% in Vietnam (5). In Eastern Africa, the pooled prevalence of pre-lacteal feeding was 12% (10) and 11.85% (11). The prevalence of pre-lacteal feeding was 40.3% in Uganda (12) and 26.8% in Kenya (13). Pre-lacteal feeding practice in Ethiopia decreased from 29% in 2005 to 8% in 2016, with a 7.2% annual rate of reduction (14). In a district-level study of Ethiopia, the magnitude of pre-lacteal feeding practice was 20.3% in Mota Tow (15), 14.2% in Debre Berhan (16), 12.6% in Jinka (17), 11.1% in North Wollo (18), 38.8% in Raya Kobo (19) and 45.4% in Harari (20). According to EDHS 2016, the national level of pre-lacteal feeding was 7.9%, and the Afar region had the highest percentage (40.7%) of pre-lacteal feeding (21). In a district-level study of the Afar region, more than half (62.5%) of the newborns had received pre-lacteal feeding within three days of birth. About 45.3% of women believe that feeding babies other than breast milk is beneficial (22). Butter, plain water, sugar solution, and milk (other than breast milk) were the common pre-lacteal foods (23–25). Animal products are highly consumed in pastoral areas where cow or goat milk is a major part of the diet for pastoralist children in addition to breastfeeding (17). The main reasons for practicing pre-lacteal feeding are the assumption of cultural taboos and beliefs (26–28), and insufficient breast milk production (18, 29, 30). Mothers with a lower socioeconomic status commonly practice pre-lacteal feeding (31). Pre-lacteal feeding was more common among mothers who were not aware of the risks (32, 33). Late initiation of breastfeeding, no counseling of breastfeeding, cesarean delivery, no antenatal care visits (1, 34, 35), no maternal education (17), and primigravida mothers (36) were risk factors for pre-lacteal feeding. On the other hand, a small family size was a protective factor for pre-lacteal feeding (37). Pre-lacteal feeding practices were associated with individual and community factors (10). Women who lived in the pastoralist region and gave birth at home were more likely to use pre-lacteal feeding (38, 39). In the Afar region, women preferred to give birth at home due to a lack of awareness about the benefits of maternity health facilities, their nomadic lifestyle, a lack of confidence and trust in health workers, and a close affinity with and easy access to traditional birth attendants (40). Another reason for home delivery in the Afar region were the cultural and spiritual factors, which included assuming delivery as a natural process that ought to happen at home, trust in traditional birth attendants, traditional practices during and after delivery, and faithfulness to religious practice, as well as traditional food preparations to be performed there (41). Despite the fact that trained traditional birth attendants have a significant impact on promoting child and maternal health in pastoralist communities, there are insufficient trained traditional birth attendants in the Afar region to meet antenatal, delivery, and postnatal care needs (42).

Pre-lacteal feeding is widely practiced in Ethiopia as a result of diverse cultural practices among Ethiopian communities; yet, there are limited studies on pre-lacteal feeding-focused research at the zonal and district levels. Particularly in the Afar region, the magnitude of pre-lacteal feeding practice and its associated factors are not studied at all. The research questions are therefore: what is the magnitude of pre-lacteal feeding practice in the Afar region? What are the contributing factors to pre-lacteal feeding practices in the Afar region? The aim of the study is to assess the magnitude of pre-lacteal feeding practice and its associated factors among mothers of children less than 2 years of age in Dubti district, Afar region, north-eastern Ethiopia. The findings generated by this study will assist in developing context-specific interventions to halt pre-lacteal feeding practices and promote optimal infant and young child feeding (IYCF) practices in the study region.

## Methods

### Study area

This study was conducted in Dubti town, which is located 20 km away in the Afar regional town of Semera, northeast Ethiopia. Most of the population in the Afar region follows Islam, and pastoralists are the main source of income. In the region, the illiteracy rate is high (43). The social systems of the Afar people are based on descent and relationships. The Afar has a patrilineal descent system that assigns a person to a certain clan (“Mela”). Children have their own herds of animals. An infant is given a female goat or a camel after birth to “look for luck.” A child is considered lucky when an animal reproduces and survives in a hazard drought. Being the first baby was an advantage in receiving more animals. Female children are usually given fewer animals than males. In Afar society, women have less social status than men. Furthermore, daughters do not inherit property equally with sons when the head of the household dies (44). Dubti is a town in northeast Ethiopia, located in Awsi Rasu zone, Afar Region. According to the Ethiopian metrology agency, this town has a latitude and longitude of 11°44′ N and 41°05′ E and an elevation of 378 meters above sea level. There are 34,310 people who live in cities, with 18,065 men and 16,245 women (45). According to the Dubti town health office data, the numbers of women of reproductive age and breastfeeding mothers were 7,372 and 2,456, respectively, at the time of data collection. The town had a total of 6,015 households, and 2,315 of those had breastfeeding mothers with children under 2 years old. The town has a health center and a referral hospital.

### Study design and sample size determination

A community-based mixed study design was used to investigate pre-lacteal feeding practice and its associated factors and to explore lived experiences of participants on pre-lacteal feeding. The quantitative study was conducted from 20 July to 25 July 2021, while the qualitative study was conducted from 26 July to 28 July 2021. The sample size for the quantitative study was calculated using the previous prevalence of pre-lacteal feeding in Dubti town in the Afar region (16.8%) (46) and 95% CI with 4% precision. The sample size was 335. By adding a 15% no response rate, the final sample size was 385. Given the low prevalence, the sample size was increased with a precision of 4% and a non-response rate of 15%. Focus group discussions (FGDs) among mothers with children under the age of two years were held in Dubti town until the qualitative data was exhausted.

### Inclusion and exclusion criteria of study participants

Biological mothers' children under the age of two years in the specified town were included in the study. Non-biological mothers, women who had not lived in the study area for six months, and mothers who were unable to talk or hear were also omitted or excluded. Mothers who had lost their children within the previous two years were excluded from the study due to the possibility of recall bias. Traditional birth attendants were interviewed as individual key informants for the qualitative study since they were the main agents in the practice of pre-lacteal feeding and had more information about it.

### Variables and operational definitions

The independent variables were selected after a thorough review of the literature. The dependent variable was pre-lacteal feeding practices, which was recorded as a binary result (yes/no).

Pre-lacteal feeding: giving any solid or liquid other than breast milk during the first three days after delivery (47).

Breastfeeding initiation time: Initiating of breastfeeding within one hour after delivery was categorized as early, whereas more than one hour latter was categorized as late or delayed (48).

### Sampling method and procedures

Quantitative data: The first step was identifying households with breastfeeding mothers who had children under the age of two years with the aid of health extension workers. A list of households (a sampling frame) was then developed, and a number was assigned to each household. The sampling interval was determined by dividing the number of households with breastfeeding mothers of under 2-year-old children (2,315) by the sample size (385), which is six. Then, a random start number was picked from the first six households, and from this first random number, households were systematically selected using the sampling interval until the calculated sample size was met. When an interviewer found two or more breastfeeding mothers with under-2-year-old children in one household, the lottery method was used. If an interviewer did not find the selected mothers with children under the age of 2 years in the first household visited, he or she visited that household repeatedly, and if the mothers were still missing, he or she moved on to the next household, where mothers with children under the age of 2 years were found.

Qualitative data: The participants were selected from mothers with children under the age of two years and key informants. Key informants were selected from traditional birth attendants using a purposive sample technique. Key informants were recruited with the help of community elders and community health extension workers. The process of selection was continued till the information was redundant. The interview participants for the qualitative study were not the same as those who were sampled for the quantitative data participants.

### Data collection instruments, methods and procedures

Closed-ended, structured interview questionnaires adapted from various literatures on pre-lacteal feeding practices were developed to collect quantitative data. The qualitative data was collected through in-depth interviews using open-ended guiding questions that were developed based on the study objective. Questionnaires were initially prepared in English and then translated into the local language (Afar). Five data collectors were trained to collect quantitative data, and two data collectors were recruited and trained to collect qualitative data. There are two supervisors: one for collecting quantitative data and the other for collecting qualitative data. All of the data collectors for both qualitative and quantitative data completed Grade 12. Before the actual data collection, 5% of the sample sizes were pre-tested outside the study area (Logyia town) using Afar language, and certain words or terms were amended after pre-testing. The purpose of the study was explained to each study participant before data collection started. Those who agreed to take part in the study were then interviewed face-to-face in a quiet and comfortable place at their home in order to better understand each other and ensure confidentiality. For the qualitative study, data collection was completed after reaching theme saturation during the interviews. The guidelines used to conduct in-depth interviews were shown in Table 1.

**Table 1:** Shows the main theme included in the in-depth interviewing guidelines.

Participants	Theme
Mothers	Types of pre-lacteal foods given to the newborn and the reason for using them.
Traditional birth attendants	Types of pre-lacteal foods given to the newborn and reason for practicing these foods.

### Data analysis

The data were entered into Epidata version 3.1 and then exported to SPSS software version 23. The study variables were described using descriptive statistics. Bivariate analysis was used to investigate the association between independent factors and pre-lacteal feeding practice. Variables with a *P*-value of less than 0.2 in the bivariate analysis were eligible for the multivariable analysis. The odds ratio of 95%CI was reported in the result. A *P*-value <0.05 was declared statistically significant. For qualitative data, the field notes were verbatim transcribed. The transcribed contents were manually read several times, then coded and grouped into categories. Finally, a theme was developed (49). The results of the thematic analysis are presented in the form of a narrative with supporting quotes. At the end, the qualitative study's findings were triangulated with the quantitative findings.

### Data quality assurance

Pre-test was done. Data collectors were chosen based on their ability to speak the local language (native speakers of the Afar language) and previous experience with data collection. Supervisors and the primary investigator double-checked each questionnaire daily to ensure there were no errors. Missing values was checked in SPSS using frequency and Cleaning was done. The field notes were translated verbatim into English by a third person who was native to the Afar language and had translation experience. Finally, the investigators double-checked the transcribed data.

### Ethical clearance

Ethical approval was obtained from a research and ethics review committee of health science college, Samara University. A letter of cooperation was given to the Dubti Town health office and town administration. Written informed consent was obtained from all the study participants, and written informed consent for all the illiterate mothers and mothers below the age of 16 years and all study children were obtained from their parents/ legal guardian(s). All methods were performed in accordance with the relevant guidelines and regulations. The right of study subjects to refuse or participate in the study at any time was respected. To preserve confidentiality, any personal identifiers were eliminated from the questionnaire. No one was harmed because of taking part in this study.

## Results

### Socio-demographic characteristics of participants

A total of three hundred seventy study subjects were recruited, with a response rate of 96.1% (370/385). About 30.3% of mothers were in the age group of 20–25 years. The children's mean age and standard deviation were 10.5 and 6.9 months, respectively, with a range of 1–23 months. Married women account for 83.5% of mothers. Housewives and Muslims have comparable percentages (62%). The majority of mothers were illiterate and of Afar ethnicity (34.9% and 47.8%, respectively). The majority of study participants earned less than 2,500 Birr per month on average (Table 2).

**Table 2:** Socio-demographic characteristics of mothers in Dubti Town, Northeast Ethiopia, in 2021.

Variable	Category	Number	Percent (%)
Mothers’ age (in years)	15–19	54	14.6
	20–25	112	30.3
	26–30	98	26.5
	31–35	59	15.9
	≥36	47	12.7
Children's mean age	10.5 ± 6.9 months (range: 1–23 months)		
Marital status	married	309	83.5
	unmarried	61	16.5
Occupation	Government employ	56	15.1
	Traders	85	23
	Housewives	229	61.9
Religious	Orthodox	98	26.5
	Muslim	229	61.9
	Protestant and catholic	43	11.6
Ethnicity	Afar	177	47.8
	Amhara	109	29.5
	Others	84	22.7
Mother education	Illiterates	133	35.9
	Primary school	120	32.4
	Secondary school	54	14.6
	College and above	63	17
Family income (Ethiopian Birr)	<2,500	120	32.4
	2,500–3,500	79	21.4
	3,501–4,500	72	19.5
	4,501–5,500	53	14.3
	>5,500	46	12.4

Others represent Oromo, Tigre, and southern nations and nationalities of people.

### Health service utilization and maternal characteristics of study participants

About 55.9% of mothers had extended family members (>4). More than half of the mothers (53.8%) had a birth interval of more than two years. Mothers with their first child accounted for 32.7%. Almost half (48.9%) of mothers had male-indexed children. About 43% of mothers did not attend an antenatal care follow-up visit. Mothers who delivered at home were 57.3%. Most of the women delivered spontaneously (Table 3).

**Table 3:** Study participants’ health service utilization and maternal characteristics in Dubti Town, Northeast Ethiopia, in 2021.

Variable	Category	Number	Percentage (%)
Number of family size	>4	207	55.9
	≤4	163	44.1
Birth interval	<2-years	171	46.2
	≥2-years	199	53.8
Birth order of indexed child	First child	121	32.7
	Subsequent child	249	67.3
Gender of indexed child	Male	181	48.9
	Female	189	51.1
Antenatal care visits	None	159	43
	1–2 times	101	27.3
	Three and above	110	29.7
Place of birth	Health institution	212	57.3
	Home	158	42.7
Mode of delivery	Cesarean section	35	9.5
	Instrumental assisted	41	11
	Spontaneous delivery	294	79.5

### Breastfeeding practices of mothers with children under the age of two years in Dubti Town, Northeast Ethiopia

Nearly 36% of mothers practiced pre-lacteal feeding. More than half of the mothers (51.6%) received breastfeeding counseling. Delayed initiation of breastfeeding accounted for 71.1% of mothers. The most pre-lacteal food was milk (15.1%), followed by water (10%) and butter (6.2%) (Table 4).

**Table 4:** Breastfeeding practices of mothers with children under the age of two years in Dubti Town, Northeast Ethiopia, in 2021.

Variables	Categories	Number	Percentage (%)
Pre-lacteal feeding practice	No	237	64.1
	Yes	133	35.9
Breastfeeding counseling	Yes	179	48.4
	No	191	51.6
Breastfeeding initiation time	Early	107	28.9
	Delayed or late	263	71.1
Types of pre-lacteal foods given to new babies	Water	37	10
	Sugar solution	17	4.6
	Butter	23	6.2
	Milk	56	15.1

### Factors associated with pre-lacteal feeding practice among mothers with children under the age of two years in Dubti Town, Northeast Ethiopia

The binary logistic regression analysis revealed that pre-lacteal feeding practice was significantly associated with the mother's education, family size, birth interval, birth order of the indexed child, gender of the indexed child, antenatal care visits, place of birth, breastfeeding initiation time, and breastfeeding counseling. When confounding factors were adjusted, being of Afar ethnicity (AOR = 2.5, 95% CI: 1.2–5.1), having an extended family size (AOR = 1.73, 95% CI: 1.02–2.9), having a birth interval of less than 2 years (AOR = 2.77, 95% CI: 1.59–4.82), the first birth order of an indexed child (AOR = 3.87, 95% CI: 2.14–7.0), male gender (AOR = 2.95, 95% CI: 1.67–5.2), no antenatal care follow-up (AOR = 2.67, 95% CI: 1.2–6.0), or once or twice antenatal care follow-up (AOR = 2.48, 95% CI: 1.14–5.4) were risk factors for pre-lacteal feeding practiced. Breastfeeding counseling (AOR = 0.38, 95% CI: 0.2–0.7) and delivery at a health institution (AOR = 0.3, 95% CI: 0.17–0.55) reduced pre-lacteal feeding practice by 62% and 70%, respectively, when compared with no breastfeeding counseling and home delivery (Table 5).

**Table 5:** Factors associated with pre-lacteal feeding practice among mothers with children under the age of 2 years in Dubti Town, Northeast Ethiopia, in 2021.

Variables	Categories	Pre-lacteal feeding		COR at 95% CI	AOR at 95% CI	*P*-value
		Yes	No			
Ethnicity	Afar	77	110	1.63 (0.96–2.79)	2.5 (1.2–5.1)*	0.016
	Amhara	29	64	1.06 (0.56–1.98)	1.14 (0.5–2.4)	0.734
	Others	27	63	1	1	1
Religious	Orthodox	39	59	1.9 (0.87–4.3)	2.42 (0.9–6.5)	0.081
	Muslim	83	146	1.65 (0.79–3.5)	1.05 (0.42–2.6)	0.913
	Protestant and catholic	11	32	1	1	1
Mothers’ education	Illiterates	54	79	2.01 (1.03–3.9)*	2.05 (0.93–4.5)	0.076
	Primary	47	73	1.9 (0.96–3.7)	2.21 (0.97–5.0)	0.058
	Secondary	16	38	1.24 (0.55–2.8)	1.19 (0.45–3.14)	0.720
	College and more	16	47	1	1	1
Family size	>4	87	120	1.84 (1.19–2.86)*	1.73 (1.02–2.9)*	0.043
	≤4	46	117	1	1	1
Birth interval	<2 years	79	92	2.31 (1.5–3.56)*	2.77 (1.59–4.82)*	<0.001
	≥2 years	54	145	1	1	1
Birth order of indexed child	First	62	59	2.64 (1.7–4.1)*	3.87 (2.14–7.0)*	<0.001
	Subsequent	71	178	1	1	1
Gender of indexed child	Males	85	96	2.6 (1.6–4.03)*	2.95 (1.67–5.2)*	<0.001
	Females	48	141	1	1	1
Ante natal care visits	None	67	92	2.24 (1.31–3.83)*	2.67 (1.2–5.95)*	0.016
	1–2 times	39	62	1.9 (1.07–3.49)*	2.48 (1.14–5.4)*	0.022
	≥3 times	27	83	1	1	1
Breastfeeding counseling	Yes	49	130	0.48 (0.31–0.74)*	0.38 (0.2–0.7)*	0.002
	No	84	107	1	1	1
Breastfeeding initiation time	Early	30	77	0.61 (0.37–0.99)*	1.2 (0.64–2.3)	0.555
	Delayed	103	160	1	1	1
Place of delivery	Health institute	63	149	0.53 (0.35–0.82)*	0.30 (0.17–0.55)*	<0.001
	Home	70	88	1	1	1

Others represent Oromo, Tigre, and southern nations and nationalities of people.

* Statistically significance, 1 = reference.

For qualitative data, a mother was purposefully selected until redundant information was reached. Seventeen participants were chosen, with four of them serving as key informants. Two of the four key informants were trained traditional birth attendants, whereas the other two were untrained birth attendants. An in-depth interview was conducted with each of them. Participants were in the age range of 19–45 years old. The Muslim religion was followed by 88.2% (15/17) of participants, while the orthodox religion was followed by the remaining participants. About 82.4% (14/17) of study participants were housewives, while 11.8% were traders. About 82.4% of the participants were illiterate and the remaining 17.6% had primary education. All the mothers who had children under the age of two years gave birth at home.

The key question posed to study participants regarding pre-lacteal foods was what you fed the newborn after delivery before beginning breastfeeding. The pre-lacteal foods found in study participants were milk products, sweet foods, water, and some plant types (Table 6).

**Table 6:** Theme, categories, and codes of the qualitative study results.

Theme	Categories	Codes
Pre-lacteal foods given to newborns	Water	Spiritual water, plain water
	Sweet foods	Sugar solution, honey
	Milk product	Milk, butter
	Plant type	Dates (Temir), condo pepper, leaf

The following study participants explained the above pre-lacteal feeding practice.

*A 34-year-old mother expressed her practical observation on pre-lacteal feeding as “after birth, testing (locally called “Ono oru”) was offered for my baby before providing breast milk.” When we asked what Ono oru was given, she replied that “the newborn must be given spiritual water (locally called “Zemzem water”), which came from Jidda” (in-depth interview with the mother)*.

*According to a 30-year-old woman's explanation, “plain water, with sugar if available, is used to clean the newborn's throat as well as serve as food. During my delivery, my breast did not secrete milk soon after birth. As a result, I gave sugar water to my baby as food and to clean his throat” (in-depth interview with the mother)*.

*A 38-year-old woman described “mostly dates (locally called Temir) and spiritual water (locally named zemzem water) were offered to the new born”. How do you give the newborn a date (Temir)? She said “first, dates (locally known as’ Temir') were added to the water, and then I gave this date (Temir) water to the newborn within a short time because of our religious order. This was passed down from the elders to the youths in our community” (in-depth interview with KII)*.

*“Camel milk was usually given to babies since it was accessible and was thought to protect them from disease. After my baby was born, my mother gave my baby camel milk (locally called ‘Han’) before breast milk. I also saw many mothers giving honey to their babies even though I did not feed honey to my infant,” a 25-year-old woman expressed (in-depth interview with the mother)*.

*A 36-year-old mother explained, “I am a traditional birth attendant, and I gave milk, butter (locally known as’ sub ah’), or sugar water to the newborn after delivery in our society. Butter is used to soften the baby's throat so that it can properly take breast milk the next time, whereas milk and sugar water are used as foods when breast milk is insufficient at the time of delivery.* *Currently, I am discontinuing this practice since training was given on the disadvantages of administering these food items prior to breastfeeding” (in-depth interview with KII)*.

*A 37-year-old woman said that “Allah allowed us to give spiritual water (locally called “zemzem water”) to the newborn to keep him healthy before giving breast milk, and my infant was also given spiritual water after delivery” (in-depth interview with mother)* .

*A 35-year-old mother reflected her practical observation after delivery was that “my child was given sugar water before breastfeeding. I knew that most mothers in our community were giving their children a small amount of either sugar water or butter because our religion ordered us to offer tests (locally called ‘onu oru’) for the newborn's post-delivery. Even though there was no sugar water available, just plain water was provided” (in-depth interview with the mother)*.

*A 33-year-old mother said, “My child was given dates (locally known as’ Temir’) water. She also pointed out that a leaf was given to the newborn after birth to prevent fear and make them brave. What kind of leaf is it? We asked. She responded, “I did not know the leaf. Traditional elders recognized this, but they couldn't tell what kind of leaf it was (in-depth interview with the mother)*.

*According to a 40-year-old mother, “my child was given spiritual* *water* *(locally called ‘zemzem water’) that came from Jidda. I've also heard from friends and others that a leaf given to a new baby helps him talkative, gentle, and hard-working during his adulthood and to take on the position of an elderly relative in the household. This leaf is secret; no one can know this leaf except the one already recognized. However, I did not give my child this leaf,” (in-depth interview with the mother)*.

*A 42-year-old woman assured us regarding a hidden leaf: “A leaf was given to the babies that allowed them to be community influential and to substitute their descend-related hero place during the adulthood period.” Could you tell us what kind of leaf it is? No, I didn't tell you. “Community elders only recognize the leaf and exclusively give it to their families, relatives, and close friends,” (In-depth interview with the mother)*.

*A 34-year-old mother described that “I had four children, and all of them were given dates (locally known as ‘Temir’) water after delivery. Dates (locally known as Temir) are considered important in our culture to prevent children from several diseases. However, health education has now been provided on the importance of exclusive breastfeeding. So, we did not give it,” (in-depth interview with the mother)*.

*A 19-year-old woman added that “since my breast did not secrete milk shortly after birth, my child was given goat milk (locally called Han). I know that most of my neighbors and relatives feed cow or goat milk to their babies when their breasts don’t produce milk,”* *(In-depth interview with the mother)*.

*A 37-years old woman expressed her practice in the community was that “testing (locally known as onu oru) could be given for the new born as soon as delivery. This was previously a common practice in our community. The testing foods could be anything in the house, such as spiritual water, goat milk (locally known as hah), butter, or a sugar solution. As a result, I had provided similar things to my baby and others in the previous year*. *However, we no longer do so since health professionals have provided training on the harmfulness of eating these foods before breast milk,” (in-depth interview with KII)*.

*A 25-year-old mother explained that “during delivery, the newborn's throat was thought to be clogged with feces, fluids, or mucus. To ensure that the breast milk was adequately taken, plain water was provided to soften and clean the newborn's throat prior to breastfeeding”, (in-depth interview with mother)*.

*A 32 year-old woman also pointed out that “condo pepper (locally called kondo berbere) was given for my infant in the nose to be harsh and hero”, (in-depth interview with mother)*.

*Another 45-year-old woman asked about condo pepper and other foods supplementation for newborn, and she said that “In the past, we used to give so many things to newborn in our community like condo pepper, leaf, and other foods. However, the society does not have interest in giving these things because the government and health professionals educated the society that giving these food items can cause disease. Therefore, the practices of taking these foods were now decreased. But, food items such as spiritual water (the local name is zemzem water), dates (locally called temir) and milk are still frequently consumed after delivery” (in-depth interview with KII)*.

*A 33-years old mother explained that “when honey is available at delivery, it is the preferred pre-lacteal meal since it has a stronger disease-prevention mechanism. However, honey was scarce in our neighborhood. As a result, milk and sugar water is frequently used as pre-lacteal meals. My child was given honey when I gave birth because I had honey in my house during delivery,” (in-depth interview with mother)*.

## Discussion

This is the first study that gives detailed information about pre-lacteal feeding practice and its associated factors among women with children under the age of 2 years in a district-level study in the Afar region of Ethiopia.

In this study, the magnitude of pre-lacteal feeding was nearly 36%. The most common pre-lacteal feedings found in this study were milk products, sweet foods, water, and some plant types. This finding is in line with the previous studies (23–25). The main reasons to give pre-lacteal feedings to children were a cultural perspective and the expectation of insufficient breast milk. These findings corroborated the findings of previous studies (18, 26–30). The causes of milk being the commonest pre-lacteal feeding in the Afar region can be attributed to the pastoralist nature of the Afar people, whose income depends on animal products. This is consistent with previous findings, which found that animal products are highly consumed in pastoralist areas where cow or goat milk is the major part of the diet for pastoralist children (17).

The current magnitude of pre-lacteal feeding practice is greater than that observed in previous studies in eastern Africa (10, 11), and Kenya (13). This discrepancy might be caused by the nomadic lives of the current study participants (40), variations in economic status (31), and limited community maternal education (43). Similarly, the current magnitude of pre-lacteal feeding was also higher than in national-level studies of the Ethiopian Demographic Health Survey (EDHS) (14, 21) and district-level studies in Ethiopia (15–18). This difference might be attributed to individual and community factors (10), including its nomadic lifestyle, a lack of trust of the community in the health workers and the benefit of health facilities, easy access to traditional birth attendants (40), cultural and spiritual factors (41), and a shortage of trained traditional birth attendants to meet antenatal, delivery, and postnatal care needs (42). However, the current magnitude is significantly lower than studies conducted in Vietnam (5), Nepal (9), and Uganda (12). Additionally, it was slightly lower than that of studies conducted in Raya Kobo (19) and Harari (20), the Afar region's district-level study (22), and the Afar regional level according to EDHS 2016 (21). This discrepancy might be attributed to the study period.

Pre-lacteal feeding was more common among people of the Afar ethnicity. This finding is consistent with the earlier findings (38, 39), which found that women who lived in the pastoral region were more likely to practice pre-lacteal feeding. One explanation for this could be that Afar women have close affinity with, trust in, and easy access to traditional birth attendants (40), faithfulness to religious practice, as well as traditional food preparation during and after delivery (41). The qualitative investigation also revealed that pre-lacteal feeding was practiced because of cultural and religious beliefs that were believed to be important for children's development and health. Pre-lacteal feeding was more common among mothers whose birth interval was less than two years. Mothers with an extended family size were more likely to practice pre-lacteal feeding. This evidence was in line with the related report's implication from the previous findings that small family size was a protective factor (35). Mothers with indexed children in their first birth order practiced more pre-lacteal feeding. This finding is consistent with a previous study that found primigravida was a risk factor for pre-lacteal feeding practice (36).The findings were explained by the notion that multiparous mothers had better knowledge and experience with breastfeeding than primiparous women (50). Pre-lacteal feeding practice was more common among male children than female children. Male birth is given due emphasis in Ethiopia, and sometimes pre-lacteal feeding is given, believing that it improves the baby's growth (51). The Afar community also places a high value on male children over female children (44), implying that gender inequality has an impact on pre-lacteal feeding practices.

Pre-lacteal feeding practices were positively associated with a lack of antenatal follow-up care or receiving it only once or twice. This finding is in line with the previous studies (34, 35). Mothers with a lack of breastfeeding counseling were more likely to practice pre-lacteal feeding than mothers with breastfeeding counseling. This evidence supports previous findings (34, 35). A lack of antenatal follow-up care or only receiving limited antenatal follow-up care could be due to high community illiteracy in the region (43), which results in no maternal education, which is associated with pre-lacteal feeding practice (17). Another reason could be ignorance of the benefits of maternity health facilities and a lack of confidence or trust in health workers (40), which lead to a lack of breastfeeding counseling that is associated with pre-lacteal feeding practice (52), as well as not being aware of the risk of pre-lacteal feeding practices (32, 33). Delivering in health institutes also helps to avoid pre-lacteal feeding practices. This finding is analogous to prior evidence that women who gave birth at home were more likely to practice pre-lacteal feedings (38, 39). In the Afar region, women preferred to give birth at home (40). The reason for preferring home delivery was trust in traditional birth attendants and traditional practices such as faithfulness to religious practice and traditional food preparations (41). Those unskilled traditional birth attendants were able to practice pre-lacteal feeding (53) because they acted as representatives of traditional practices, beliefs, and expectations (41).

The limitation of the study is that it is unable to infer cause-effect relationships and the information received from mothers could be subjected to recall bias. Using a mixed study design helps triangulation and gains additional information not found in the quantitative study. The study's findings have a significant impact on the promotion of optimal breastfeeding practices and achieving sustainable development goal of decreasing child mortality in the study areas. These findings will be used to develop a regional intervention strategy.

## Conclusion

A significant number of study participants practiced pre-lacteal feeding. Afar ethnicity, an extended family size, a birth interval of less than 2 years, the first birth order of an indexed child, male-indexed children, and no or only one or two antenatal visits were factors associated with pre-lacteal feeding practice. The community should receive a public health education program that emphasizes the reduction of extended families and birth intervals, as well as the equality of children based on their gender and birth order. Antenatal care follow-up also needs to be reinforced, and the community and primary health care facilities need to be made aware of its significance. Breastfeeding counseling and the uptake of delivery in a health facility should be increased and strengthened. Dairy products, water, and sweet foods were the most commonly used pre-lacteal feeding practices. The cultural belief and the assumption of insufficient breast milk were the key reasons for practicing pre-lacteal feeding in the study area. It is essential to provide culturally appropriate education about the drawbacks of pre-lacteal feeding in order to resolve conflicts between cultural or religious beliefs and the adverse effects of pre-lacteal feeding because women culturally value pre-lacteal feeding for a child's health and development.

## Data Availability

The raw data supporting the conclusions of this article will be made available by the authors, without undue reservation.
